# Extensive Neuronal Differentiation of Human Neural Stem Cell Grafts in Adult Rat Spinal Cord

**DOI:** 10.1371/journal.pmed.0040039

**Published:** 2007-02-13

**Authors:** Jun Yan, Leyan Xu, Annie M Welsh, Glen Hatfield, Thomas Hazel, Karl Johe, Vassilis E Koliatsos

**Affiliations:** 1 Department of Pathology, Division of Neuropathology, The Johns Hopkins Medical Institutions, Baltimore, Maryland, United States of America; 2 Neuralstem, Rockville, Maryland, United States of America; 3 Department of Neurology, The Johns Hopkins Medical Institutions, Baltimore, Maryland, United States of America; 4 Department of Neuroscience, The Johns Hopkins Medical Institutions, Baltimore, Maryland, United States of America; 5 Department of Psychiatry and Behavioral Sciences, The Johns Hopkins Medical Institutions, Baltimore, Maryland, United States of America; Albany Medical College, United States of America

## Abstract

**Background:**

Effective treatments for degenerative and traumatic diseases of the nervous system are not currently available. The support or replacement of injured neurons with neural grafts, already an established approach in experimental therapeutics, has been recently invigorated with the addition of neural and embryonic stem-derived precursors as inexhaustible, self-propagating alternatives to fetal tissues. The adult spinal cord, i.e., the site of common devastating injuries and motor neuron disease, has been an especially challenging target for stem cell therapies. In most cases, neural stem cell (NSC) transplants have shown either poor differentiation or a preferential choice of glial lineages.

**Methods and Findings:**

In the present investigation, we grafted NSCs from human fetal spinal cord grown in monolayer into the lumbar cord of normal or injured adult nude rats and observed large-scale differentiation of these cells into neurons that formed axons and synapses and established extensive contacts with host motor neurons. Spinal cord microenvironment appeared to influence fate choice, with centrally located cells taking on a predominant neuronal path, and cells located under the pia membrane persisting as NSCs or presenting with astrocytic phenotypes. Slightly fewer than one-tenth of grafted neurons differentiated into oligodendrocytes. The presence of lesions increased the frequency of astrocytic phenotypes in the white matter.

**Conclusions:**

NSC grafts can show substantial neuronal differentiation in the normal and injured adult spinal cord with good potential of integration into host neural circuits. In view of recent similar findings from other laboratories, the extent of neuronal differentiation observed here disputes the notion of a spinal cord that is constitutively unfavorable to neuronal repair. Restoration of spinal cord circuitry in traumatic and degenerative diseases may be more realistic than previously thought, although major challenges remain, especially with respect to the establishment of neuromuscular connections.

## Abstract 2

When neural stem cells from human fetal spinal cord were grafted into the lumbar cord of normal or injured adult nude rats, substantial neuronal differentiation was found.

## Editors' Summary

**Background.:**

Every year, spinal cord injuries, many caused by road traffic accidents, paralyze about 11,000 people in the US. This paralysis occurs because the spinal cord is the main communication highway between the body and the brain. Information from the skin and other sensory organs is transmitted to the brain along the spinal cord by bundles of neurons, nervous system cells that transmit and receive messages. The brain then sends information back down the spinal cord to control movement, breathing, and other bodily functions. The bones of the spine normally protect the spinal cord but, if these are broken or dislocated, the spinal cord can be cut or compressed, which interrupts the information flow. Damage near the top of the spinal cord can paralyze the arms and legs (tetraplegia); damage lower down paralyzes the legs only (paraplegia). Spinal cord injuries also cause many other medical problems, including the loss of bowel and bladder control. Although the deleterious effects of spinal cord injuries can be minimized by quickly immobilizing the patient and using drugs to reduce inflammation, the damaged nerve fibers never regrow. Consequently, spinal cord injury is permanent.

**Why Was This Study Done?:**

Scientists are currently searching for ways to reverse spinal cord damage. One potential approach is to replace the damaged neurons using neural stem cells (NSCs). These cells, which can be isolated from embryos and from some areas of the adult nervous system, are able to develop into all the specialized cells types of the nervous system. However, because most attempts to repair spinal cord damage with NSC transplants have been unsuccessful, many scientists believe that the environment of the spinal cord is unsuitable for nerve regeneration. In this study, the researchers have investigated what happens to NSCs derived from the spinal cord of a human fetus after transplantation into the spinal cord of adult rats.

**What Did the Researchers Do and Find?:**

The researchers injected human NSCs that they had grown in dishes into the spinal cord of intact nude rats (animals that lack a functioning immune system and so do not destroy human cells) and into nude rats whose spinal cord had been damaged at the transplantation site. The survival and fate of the transplanted cells was assessed by staining thin slices of spinal cord with an antibody that binds to a human-specific protein and with antibodies that recognize proteins specific to NSCs, neurons, or other nervous system cells. The researchers report that the human cells survived well in the adult spinal cord of the injured and normal rats and migrated into the gray matter of the spinal cord (which contains neuronal cell bodies) and into the white matter (which contains the long extensions of nerve cells that carry nerve impulses). 75% and 60% of the human cells in the gray and white matter, respectively, contained a neuron-specific protein six months after transplantation but only 10% of those in the membrane surrounding the spinal cord became neurons; the rest developed into astrocytes (another nervous system cell type) or remained as stem cells. Finally, many of the human-derived neurons made the neurotransmitter GABA (one of the chemicals that transfers messages between neurons) and made contacts with host spinal cord neurons.

**What Do These Findings Mean?:**

These findings suggest that human NSC grafts can, after all, develop into neurons (predominantly GABA-producing neurons) in normal and injured adult spinal cord and integrate into the existing spinal cord if the conditions are right. Although these animal experiments suggest that NSC transplants might help people with spinal injuries, they have some important limitations. For example, the spinal cord lesions used here are mild and unlike those seen in human patients. This and the use of nude rats might have reduced the scarring in the damaged spinal cord that is often a major barrier to nerve regeneration. Furthermore, the researchers did not test whether NSC transplants provide functional improvements after spinal cord injury. However, since other researchers have also recently reported that NSCs can grow and develop into neurons in injured adult spinal cord, these new results further strengthen hopes it might eventually be possible to use human NSCs to repair damaged spinal cords.

**Additional Information.:**

Please access these Web sites via the online version of this summary at http://dx.doi.org/doi:10.1371/journal.pmed.0040039.

- The US National Institute of Neurological Disorders and Stroke provides information on spinal cord injury and current spinal cord research
- Spinal Research  (a UK charity) offers information on spinal cord injury and repair
- The US National Spinal Cord Injury Association Web site contains factsheets on spinal cord injuries
- MedlinePlus encyclopedia has pages on spinal cord trauma and interactive tutorials on spinal cord injury
- The International Society for Stem Cell Research  offers information on all sorts of stem cells including NSCs
- The US National Human Neural Stem Cell Resource provides information on human NSCs, including the current US government's stance on stem cell research

## Introduction

Degenerative and traumatic diseases of the nervous system are characterized by loss of neurons and their connections. Effective treatments for these conditions are presently unavailable. In the field of experimental therapeutics two major approaches have been taken: prevention of cell death with compounds that interfere with decision-making steps in cell death pathways and the replacement or support of degenerating neurons with neural grafts [1,2]. Neural stem cells (NSCs) are a promising alternative to fetal tissues for cell therapies, primarily due to their self-renewal and pluripotentiality [3,4]. The use of human-derived NSCs has the additional advantage of yielding translational information that is relevant to clinical therapeutics.

The adult spinal cord represents an especially challenging environment for the survival and differentiation of NSCs because of the apparent lack of cells and/or signals promoting regeneration [5]. For the most part, NSC grafts into the adult injured cord have shown either poor differentiation [6,7] or a restricted differentiation into the glial lineage [8,9], the latter attributable to the relative preference of pluripotent precursors for non-neuronal fates. However, a few studies using embryonic stem cell-/embryonic body-derived NSCs [10] or lineage-restricted neuronal progenitors [11,12] have achieved good neuronal differentiation of grafted cells, thus mounting a challenge to the notion of the spinal cord as an environment unfavorable to neuronal differentiation. In the present report, we grafted NSCs derived from human embryonic cord and grown in monolayer into the spinal cord of normal or injured adult rats and explored the differentiation fate and degree of structural integration of these cells as a function of time, anatomical location, and presence or absence of injury.

## Methods

### Derivation of Human NSCs

Human NSCs were prepared from the cervical and upper thoracic spinal cord of a single eight-week human fetus after an elective abortion. The tissue was donated by the mother in a manner fully compliant with the guidelines of the National Institutes of Health (NIH) and the Food and Drug Administration (FDA) and approved by an outside independent review board. Spinal cord tissue was cleared of meninges and dorsal root ganglia and dissociated into a single-cell suspension by mechanical trituration in serum-free, modified N2 medium composed of 100 mg/l human plasma apo-transferrin, 25 mg/l recombinant human insulin, 1.56 g/l glucose, 20 nM progesterone, 100 μM putrescine, and 30 nM sodium selenite in DMEM/F12, to which basic fibroblast growth factor (bFGF) (10 ng/ml) was added. The initial culture was serially expanded as monolayers in precoated flasks or plates as described [13]. Approximately 6.1 × 10^6^ cells were obtained upon the initial dissociation of the spinal cord tissue. All cells were plated onto one 150-mm plate in 20 ml of growth media. Growth medium was changed every other day and, on alternate days, 10 ng/ml of bFGF was added.

The first passage was conducted at 16 d postplating. At this time point, the culture was composed mostly of dividing NSCs and postmitotic neurons. Dividing cells were harvested by brief treatment with trypsin (0.05% + 0.53 mM EDTA) followed by mechanical dissociation. A single-cell suspension was thus derived that was centrifuged at 1,400 rpm for five minutes. The cell pellet was resuspended in the growth medium, and cells were replated in new precoated plates at 1.2 × 10^6^ cells in 20 ml of medium per 150 mm plate. Cells were harvested at approximately 75% confluence, which occurred within five or six days. This process was repeated for 20 passages. Cells from various passages were frozen in the growth medium plus 10% DMSO and stored in liquid nitrogen. Upon thawing, overall rate of recovery was 80%–95%. The resulting cell line, produced by epigenetic means only and by using bFGF as the sole mitogen, was coded “566RSC.”

Passage 10–12 cells were used in this study. One cryopreserved vial of the appropriate passage was thawed, washed, and cultured again as described above, 5–7 d prior to surgery. For multiple days of surgeries, cultures were seeded at varying densities, so that each flask reached confluence on the designated day of surgery. Cells were subsequently harvested by brief enzymatic treatment as described above, washed in a buffered saline solution, couriered to the surgery site on wet ice, and used within 24 h. Viability of cells on ice was typically greater than 80% within this 24-h period.

### Experimental Subjects and Surgical Procedures

Nude rats (160–180 g, strain CR: NIH-RNU, *n* = 37) were purchased from the animal service of the National Cancer Institute and were the main experimental subjects of this study. These animals were subjected to surgical interventions 2 wk prior to NSC grafting; interventions included proximal ventral rhizotomies (*n* = 18) [14], excitotoxic lesions (*n* = 12) [15], or sham surgeries (*n* = 6). An additional small number of commonly used Sprague-Dawley rats (*n* = 3, Charles River Laboratories [http://www.criver.com]) was used for the purpose of proving the concept that the results reported here were unrelated to the athymic state of our experimental animals. These animals were used for transplantation without prior lesions and were immunosuppressed with daily injections of FK506 (2 μg/g i.p., Prograf, Fujisawa Healthcare [http://www.astellas.com/]). Surgeries were performed using gas anesthesia (enflurane:oxygen:nitrous oxide = 1:33:66) and aseptic methods. Animal surveillance and the surgical procedures described here were carried out according to protocols approved by the Animal Care and Use Committee of the Johns Hopkins Medical Institutions.

Rhizotomies involved transections of L4 and L5 roots with extraspinal avulsion of the corresponding spinal nerves as described [14]. In brief, the left L4 and L5 spinal nerve roots were exposed at the level of the iliac crest after splitting the sacroiliac joint, and roots were avulsed by applying a steady moderate traction with forceps. Excitotoxic lesions were made with 100 μl of 150 mM of the kainate analog L-homocysteic acid (HCA) [15] absorbed into a gelfoam pledget that was laid on the dorsal pia of L4–L5 for a period of 20 min after a dorsal laminectomy. This strategy optimizes the need to lesion a rather extensive group of vulnerable (motor) neurons throughout L4–L5 without causing epileptogenesis. The sham group was subjected to dorsal laminectomy and subsequent wound closure. The parallel use of a severe, proximal axotomy lesion and a mild neurotoxic injury allowed for some experimental variance in the method and degree of motor neuron injury; the neurotoxic paradigm offers the additional advantage of preserving the conduit for the assessment of de novo axonal elongation toward the periphery. Of note, both types of lesions involve minimal disruption of the blood-brain barrier and, therefore, considerably reduce the likelihood of glial scarring.

Animals were subjected to dorsal laminectomy at the lower thoracic level and received four injections of 10^5^ NSCs each in 0.5 μl suspension into ventral L4 and L5 on the left side 2 wk after lesion. Injections were made stereotaxically on a Kopf spinal unit 1 mm lateral to the midline using pulled-beveled glass micropipettes connected, via silastic tubing, to 10 μl Hamilton microsyringes. Animals were allowed to survive for 3 wk, 3 mo, or 6 mo (avulsion lesions), 3 or 6 mo (excitotoxic lesions), or 6 mo (sham lesions) (*n* = 6 for each treatment × time point).

### Histology, Immunocytochemistry, and Microscopy

The survival and phenotypic fate of NSCs were assessed with immunocytochemistry (ICC), including ABC-peroxidase ICC and dual-label immunofluorescence. Tissues were prepared from animals perfused with 4% freshly depolymerized, neutral-buffered paraformaldehyde. The thoracolumbar spinal cord segments with attached roots and lumbar nerves were further fixed by immersion in the same fixative for an additional 4 h after removal of the dura. Blocks containing the entire grafted area plus 1 mm border above and below were subdissected, equilibrated in 30% neutral-buffered sucrose, and frozen for further processing. L3–S1 roots were processed separately as whole-mount preparations or after teasing the rootlets with heat-coagulated tips of glass pipettes. Blocks were sectioned transversely (30 μm) on a freezing microtome; sections were kept in an antifreeze solution until processed for NSC survival or phenotypic studies. Survival studies utilized human nuclear antigen (HNu) immunoperoxidase-stained sections (~ 15 per animal, i.e. every 24th section through the L3–S1 block) with random sampling of the first section. HNu is a selective nuclear marker of cells of human origin [16]. NSC differentiation utilized four to five sections 1.5 mm apart taken through the grafting area and stained with dual-label immunofluorescence; in most cases, dual-label immunofluorescence combined HNu with another cellular marker.

After permeabilization with 0.1% Triton X-100 and nonspecific site blocking with 5% normal serum, slide-mounted sections were incubated in primary antibodies in 1 mg/ml BSA with 0.1% Triton X-100 (4 °C, overnight). Primary antibodies were used to address human (graft) versus rat (host) cell identity, mitotic activity, and neuronal, astrocytic, and oligodendrocytic phenotype specification and included a number of monoclonal antibodies and antisera as laid out in Table 1. Normal immunoglobulin G (IgG) from the species of origin of the primary antibodies served as negative controls. Peroxidase-based detection of HNu immunoreactivity utilized an enhanced version of the avidin-biotin method (ABC-elite kit; Vector [http://www.vectorlabs.com/]) and a standard DAB chromagen reaction. Dual immunofluorescence utilized an indirect protocol combining goat/donkey anti-mouse IgG and goat/donkey anti-rabbit IgG labeled with Cy3 or Cy2 in a corresponding fashion (1:200; Jackson ImmunoResearch [http://www.jacksonimmuno.com/]); anti-rabbit IgGs were used against the species of origin of the phenotype-marking antibody. Sections were incubated in these antibodies for 2–4 h at RT and were then counterstained with the fluorescent DNA dye DAPI. Sections were mounted with DPX and studied with epifluorescence or confocal microscopy. Triple immunofluorescence was used for the colocalization of HNu, Bsn (Bassoon), and TUJ1. This procedure involved first a dual (indirect) immunofluorescent step, in which Cy5 replaced Cy3 as fluorophore for the goat anti-mouse IgG, and then a direct immunofluorescent step, in which sections were incubated in HNu antibody coupled to the red fluorophore Alexa 594 (Molecular Probes [http://probes.invitrogen.com/]).

**Table 1:**
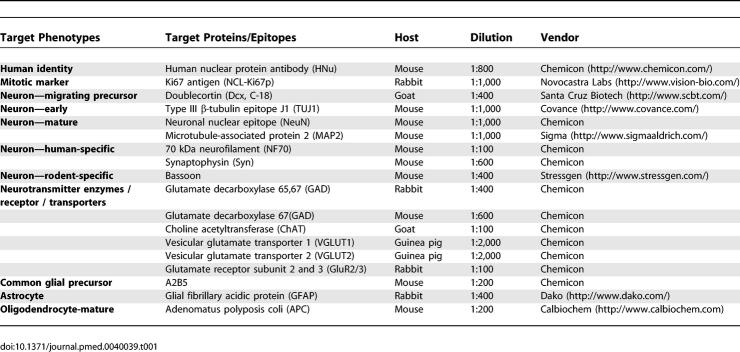
Primary Antibodies

### Cell Counts for Surviving and Differentiating NSCs

To assess NSC graft survival, we counted numbers of HNu (+) profiles through L4–L5 using stereological assumptions based on the optical fractionator concept [17]. We used serial immunoperoxidase-stained sections that were sampled in a randomized systematic fashion. Corrected section thickness after processing was 18 μm. The optical fractionator probe was applied with the aid of a motorized-stage Axioplan microscope (Zeiss, www.zeiss.de) equipped with the Stereo Investigator V hardware and software (MicroBrightField [http://www.mbfbioscience.com/]). Contours of half spinal cords were outlined at 5× by a blinded investigator, and cells were counted using a 100× oil-immersion objective. A 40 × 35 μm counting frame was used within a 300 μm × 300 μm grid coextensive with the outlined area. Counting depth (optical dissector height) ranged 9–13 μm depending on average section thickness. A guard volume of 1.0 μm was used to avoid sectioning-related artifacts such as lost caps and uneven section surfaces.

To study NSC differentiation, we used a nonstereological method of counting total number of HNu (+) cells, as well as cells dually labeled with HNu and a phenotypic marker on randomly selected 100× fields through the ventral horn from immunofluorescent preparations. One field in each one of four sections, spaced 1 mm apart through the grafting area, was used from each animal. Numbers of HNu (+) and double-labeled profiles were pooled from all four fields counted from each case and grouped per experimental protocol. NSCs located at or near the meninges were counted separately. Percentages of double-labeled cells were generated per parenchymal or meningeal sites at different time points for each treatment group (*n* = 4 animals per group). In the sham group we only examined 6-mo data, and in the HCA group data were available from the 3- and 6-mo, but not the 3-wk group. On average, 2,040 and 1,970 cells were counted per subject in parenchymal and meningeal sites, respectively. In the 6-mo group, a more detailed anatomical analysis of regional differentiation of grafted NSCs was performed. In these cases, separate numbers were generated in the ventral horn, dorsal horn, and ventral white matter, as outlined in the Results section.

Variation in survival and differentiation, as function of experimental protocol, graft location, and time point postsurgery, was studied with one-way ANOVA followed by Tukey's multiple comparison post hoc test. Results are expressed as mean ± standard deviation.

### Real-Time PCR Analysis of the Expression of Relevant Trophic and Tropic Genes

RNA samples were extracted from 10^7^ NSCs at various time points of differentiation in vitro (0, 14, 29, and 42 d after withdrawal of bFGF) using Trizol (Invitrogen [http://www.invitrogen.com/]). Residual DNA was removed using the DNA-free kit (Ambion [http://www.ambion.com/]). Template cDNA was synthesized from 1 μg of RNA with the iScript cDNA Synthesis kit (Bio-Rad http://www.bio-rad.com/]) and was diluted 10-fold before PCR amplification. SYBR green-based real-time PCR was performed using the iCycler (Bio-Rad). 18s rRNA was used as the reference transcript (e.g., control gene). Table 2 lists the human-specific primers for bFGF [18], brain-derived neurotrophic factor (BDNF) [19], vascular endothelial growth factor (VEGF) [18], glial cell line-derived neurotrophic factor [20], insulin-like growth factor-1 (IGF-1) [21], and neuregulins (NRGs) 1, 2, 3 (NRG 1, 2, and 3) [22], all of which were assayed because of a suspected or established role in motor neuron development. PCR reactions, run in triplicate for each sample, were carried out in 25 μl volume containing 2 μl of 1:10 dilution of cDNA, 0.5 μl of each sense and antisense 10 μM primer stocks, 12.5 μl iQ SYBR green Supermix (Bio-Rad), and nuclease-free water. For either 18s rRNA or mRNA of interest, except NRG transcripts, cycling conditions were 95 °C for 5 min, followed by 40 cycles of 95 °C for 15 s and 60 °C for 1 min. For NRG 1, 2, and 3 the annealing temperature was 56 °C. Reactions without template served as negative controls. Melting curve analysis was carried out by heating the amplicon from 50 °C to 95 °C in 90 0.5 °C increments. PCR efficiency curves were made for each gene using five duplicate 5-fold dilutions, corresponding to 0.08 to 50 ng of the initial total RNA input into the RT reaction. Data were analyzed using the Bio-Rad Gene Expression Macro version 1.1 for Microsoft Excel. Normalized gene expression ratios were calculated as described [23]. Briefly, PCR efficiencies were calculated according to the equation *E* = *e* ^[−1/slope]^. The relative expression ratio of target genes was calculated using the equation: ratio = (*E*_target_)^Δ target Ct (control-sample)^/(*E*_ref_)^Δ reference Ct (control-sample)^, where the Ct is the point at which SYBR green fluorescence rises above background fluorescence. Gene expression levels from Day 0 cDNA were designated as 1, to which other time points were compared. Standard deviations for the relative and normalized expression values were calculated as described [24].

**Table 2:**
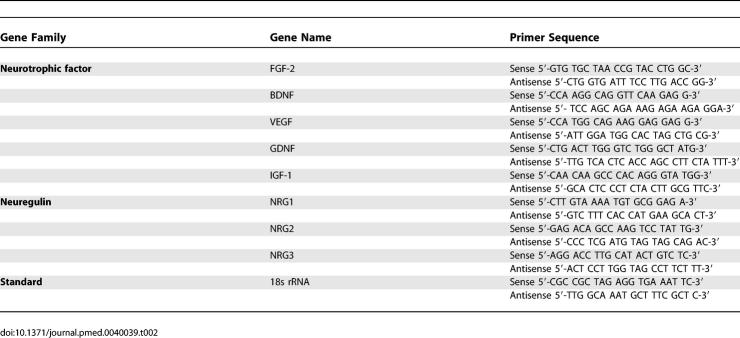
Primer Sequences Used for Real-Time PCR

## Results

### In Vitro Differentiation and Trophic/Tropic Profile of Human NSCs

**Figure 1:**
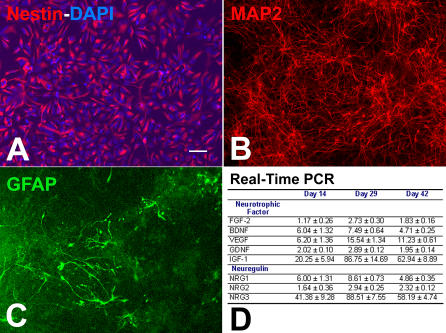
In Vitro Differentiation of Human NSCs Used for Transplantation (A) The vast majority of cells express the NSC-specific marker nestin (red) immediately before grafting. The DNA dye DAPI (blue) was used to reveal all cells in culture. (B and C) At 14 days within the differentiation phase (i.e., after bFGF removal), ~ 50% of cells acquire MAP2 immunoreactivity and neuronal cytology, with characteristic processes (red, B). A smaller number of cells differentiate into GFAP (+) astrocytes (green, C). (D) Real-time RT-PCR data showing increased neurotrophic factor and NRG expression in the course of NSC differentiation in vitro. The number of days on top of the columns is the days NSCs have been in a phase of differentiation (after withdrawal of fibroblast growth factor). Results are expressed as fold increases compared to levels expressed at the proliferation phase (day 0), the latter values designated as 1. Data represent average ± standard deviation of triplicate measurements of a representative cell culture sample at a given time point. The experiment was repeated twice with different sets of cell samples and yielded very similar results. Scale bars: 50 μm.

NSCs prepared for grafting were propagated as a monolayer culture in the presence of bFGF and delivered to animals within 24 h post-bFGF withdrawal. At that time, all cells expressed the NSC marker nestin (Figure 1A), approximately 5% were immunoreactive for the neuronal precursor-specific marker PSA-NCAM, and less than 1% expressed the neuronal markers TUJ1 and MAP2 or the astroglial marker glial fibrillary acidic protein (GFAP). With continued culture in the absence of bFGF, about 50% of cells acquired neuronal phenotypes as shown by MAP2 immunoreactivity and cytological profile (Figure 1B), and some differentiated into GFAP (+) astrocytic profiles (Figure 1C) within 2 wk. Very few cells expressed oligodendrocyte/Schwann cell markers within this time frame.

The expression of representative neurotrophic factors and NRGs was studied by real-time PCR at 0, 14, 29, and 42 days post-bFGF withdrawal, i.e. during the differentiation phase (Figure 1D). Among neurotrophic factors, glial cell line-derived neurotrophic factor and BDNF transcripts increased 3- and 7-fold respectively, whereas VEGF increased 15-fold by day 29; significant sustained increases were also noted for IGF-1 (Figure 1D). Among NRGs, NRG1 and NRG3 transcripts, particularly the latter, were also found to have significantly increased. The expression of all these factors declined after 4 wk, a pattern that may reflect some cellular deterioration that is common in long-term culturing of mature neurons.

### Survival and Migration of Human NSCs in Rat Spinal Cord

**Figure 2:**
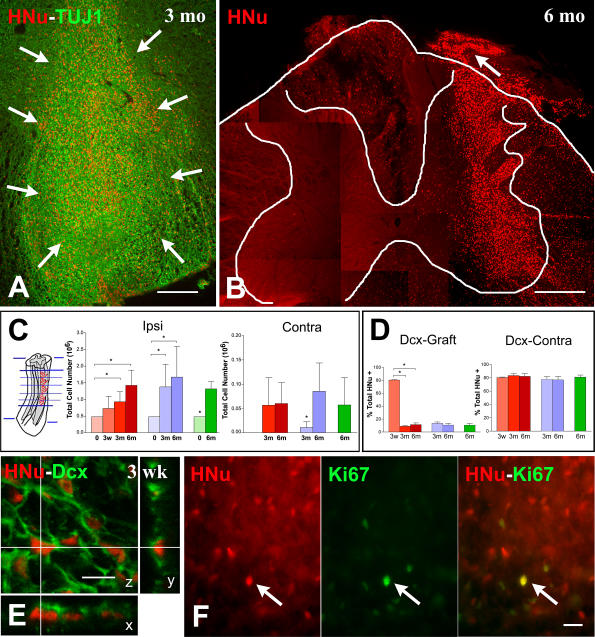
Survival and Migration of Human NSCs in Rat Spinal Cord Photomicrographs and graphs in (A–C) illustrate the localization and numbers of HNu (+) cells at different time points postgrafting, whereas (D) and (E) support the migratory phenotype of grafted HNu (+) cells, and (F) confirms their low mitotic activity. (A and B) At 3 mo postgrafting most HNu (+) cells, indicated as red profiles with an arrow in (A), are located around the injection sites and along needle tracks. By six months (B), HNu (+) cells show widespread migration away from the injection site in both the gray and white matter, and many are seen in the white matter and a few in the gray matter of the contralateral side. (B) is a composite of several fields to show the extent of migration. Arrow in (B) shows the colonization, by NSC-derived cells, of the central nervous system portion of the dorsal root (note the central nervous system–peripheral nervous system transition zone). (C) Bar graphs showing HNu (+) cell numbers at the time of grafting (0) and at three weeks (3w), three months (3m), and six months (6m) postgrafting in the different treatment groups (avulsion, red; HCA treatment, blue; sham, green). Far left graph shows numbers of HNu (+) cells ipsilateral to the grafting site (Ipsi), and far right graph shows numbers on the contralateral gray matter (Contra). Brackets show the results of post hoc testing when ANOVA was significant in the avulsion and HCA groups ipsilateral to grafting; in all other cases, significance was established with a Student's t-test. Asterisk indicates statistical significance at *p* ≤ 0.05. Method of section selection is illustrated on the extreme left. (D) Dcx, a marker for migrating neuronal precursors, was expressed by about 80% of grafted cells 3 wk postgrafting. Dcx expression is reduced to 10%–15% of HNu (+) cells surrounding the grafting sites at 3 and 6 mo but remains very high (~ 80%) in HNu (+) cells on the contralateral gray matter up to 6 mo postgrafting. (E) A confocal image of HNu (+) (red) cells also labeled with Dcx (green) at 3 wk postgrafting. (F) The three images illustrate, on a section that was dually stained for HNu (red nuclear marker on the left) and Ki67 (green nuclear marker in the center), the very low rate of mitotic activity (double-stained nuclei on the right) in NSC grafts. The single double-stained nuclear profile is indicated with an arrow. Scale bars: (A) 200 μm; (B) 600 μm; (E) 10 μm; (F) 20 μm.

The examination of all stained sections from lesioned and control animals shows a robust engraftment and excellent long-term survival of NSCs in the adult spinal cord environment (Figure 2A and 2B). Stereological estimates of surviving HNu (+) cells show an average of 1.5 × 10^6^ cells in the L4–L5 cord 6 mo postgrafting. This value represents a 3- to 4-fold increase of the cell population present in the initial graft (Figure 2C) and implies that the initial graft survived well and underwent, on average, two mitotic divisions. This estimate is consistent with a low frequency (3%–5%) of HNu (+) cells that also expressed the nuclear antigen Ki67 at 3 and 6 mo postgrafting in all experimental conditions studied in this paper (Figure 2F). Ki67 is a marker of all phases of cell cycle minus G0. and Ki67-positive cells were found randomly dispersed across the graft area without evidence of clustering in specific sites.

A comparison among animals surviving for 3 wk, 3 mo, and 6 mo shows a tendency of NSCs to migrate away from the initial grafting sites and populate both gray and white matter, as well as the proximal end of ventral and dorsal roots in the avulsion cases. Approximately 3% of NSC-derived cells were found in the contralateral side. There were more cells in the contralateral side in avulsion-injured than in HCA-injured animals three months postgrafting (Figure 2C, far right graph). The migratory disposition of NSCs was further confirmed with the expression of doublecortin (Dcx), a microtubule-associated protein that serves as a specific marker for migrating neuronal precursors [25], by about 80% of grafted cells three weeks postgrafting (Figure 2D and 2E). Dcx expression is reduced to 10%–15% of HNu (+) cells at the grafting sites at 3 and 6 mo but remains very high (~ 80%) in HNu (+) cells on the contralateral side at 6 mo postgrafting, evidence for continuous migration (Figure 2D).

In summary, human NSCs survive very well in the spinal cords of nude rats with minimal further mitotic activity irrespective of the presence or absence of lesions, and migrate extensively into the ipsilateral and contralateral spinal cord.

### Differentiation of Human NSCs into Neuronal and Non-Neuronal Cells: Parenchyma versus Meninges, Gray versus White Matter

**Figure 3:**
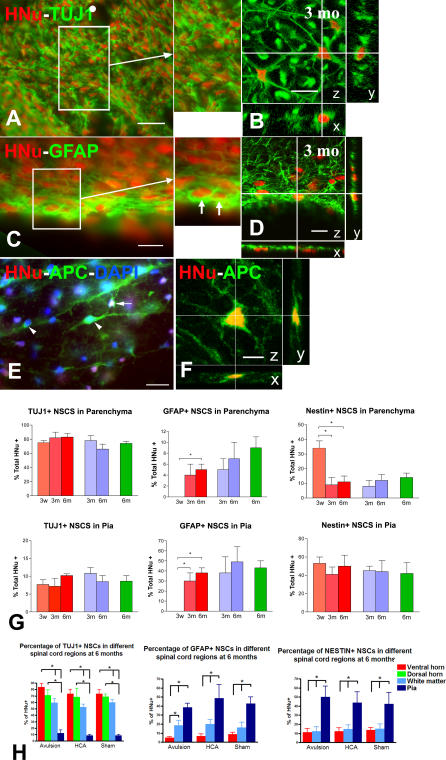
Differentiation of Grafted Human NSCs into Neurons and Glial Cells Photomicrographs (A–F) illustrate cases of neuronal (A and B), astrocytic (C and D), and oligodendrocytic (E and F) differentiation of HNu (+) cells by epifluorescence (A, C, and E) or confocal (B, D, and F) microscopy. (G) is a composite of bar graphs illustrating the general differences in fate choice between parenchymal (upper level) and meningeal (lower level) sites of NSC grafts. (H) provides further detail in differential fate choice among three parenchymal sites and the pia compared side-by-side. (A and B) These two sections are stained for HNu and TUJ1 and show the abundance of NSC-derived neurons within the parenchyma of the ventral horn by epifluorescence (A) and confocal microscopy (B). Both preparations are taken from animals killed three months postgrafting. Inset is a magnification of demarcated area in (A). Note the homogeneous appearance of TUJ1 (+) cells in the A inset. Confocal sections have been virtually resectioned at the *x* and *y* planes to confirm the identity of the double-stained structure. (C and D) These sections, dually stained for HNu and GFAP, illustrate the substantial astrocytic differentiation of NSCs located by the pia membrane by (Figure 3, continued) epifluorescence (C) and confocal microscopy (D). Inset is a magnification of demarcated area in (C), and representative astrocytes are indicated with arrows. Confocal sections have been processed as in (B). (E and F) Oligodendrocyte differentiation in ventral white matter based on APC immunoreactivity in the cytoplasm of cells with HNu (+) nuclei as shown with epifluorescence (E) and confocal microscopy (F). Blue nuclei represent DAPI counterstain. Arrow depicts a double-labeled cell. Arrowheads point to host oligodendrocytes (APC [+], HNu [−] cells). Confocal sections have been processed as in (B). (G) Bar graphs depicting the fate choices of NSC grafts in the parenchyma (including ventral and dorsal horn and ventral white matter, upper graphs) or the meninges (lower graphs) at three weeks (3w), three months (3m), and six months (6m) in different treatment groups (avulsion, red; HCA treatment, blue; sham, green). Neuronal fate is represented by numbers of TUJ1-labeled HNu (+) cells, and astrocytic fate is represented by numbers of GFAP-labeled HNu (+) cells. NSCs in a neural stem/precursor state are depicted here as nestin-and HNu double-labeled cells. Asterisks indicate critical post hoc differences between subgroups where ANOVA is significant (*p* ≤ 0.05). (H) These graphs provide further detail into the role of spinal microenvironment in the fate choice of grafted NSCs by differentiating among three parenchymal sites and the pia. Cell fates are represented by the same markers as in (G) Asterisks on top of brackets indicate important post hoc differences where ANOVA is significant (*p* ≤ 0.05). Scale bars: (A), (C), (E) 20 μm; (B), (D), (F) 10 μm.

Based on the aiming of the grafting pipettes, the main portion of NSC grafts reviewed here was confined within the ventral horn of L4–L5 segments (Figure 2A). In this location, the majority of grafted cells entered a neuronal lineage, as evidenced by a ≥ 75% rate of TUJ1 immunoreactivity at 3 wk and at 3 and 6 mo postgrafting (Figure 3A, 3B, and 3G). These TUJ1 (+) cells have round or bipolar cell bodies and an average diameter of 10 μm (Figure 3A and 3B). Rates of TUJ1 differentiation did not differ significantly among treatment groups and were consistent with results from dual ICC for HNu and the neuronal nuclear epitope NeuN. In addition, there were no significant differences in rates of TUJ1 (+) NSC-derived cells among various time points, evidence that the establishment of a neuronal lineage occurs very early in the life of these grafts. Compared to neuronal markers, the appearance of astrocytic phenotypes was slower. HNu (+) cells do not show significant GFAP immunoreactivity at 3 wk postgrafting. By 3–6 mo, ~ 5% of grafted HNu (+) cells in the avulsion cases and a slightly higher proportion of cells in HCA (−) and sham animals stained for GFAP (Figure 3G). The astrocytic cytology of these GFAP (+) cells is confirmed by confocal microscopy (Figure 3D). Nestin expression in grafted cells declined over the time course studied here and the greatest reduction was noted between 3 wk and 3 mo. A percentage of 11%–14% of HNu (+) cells remained nestin (+) even at 6 mo postgrafting (Figure 3G).

The fate of grafted human NSCs located in dorsal horn exhibited similar patterns as in ventral horn. At 6 mo postgrafting, rates of TUJ1 (+) NSC-derived cells in dorsal horn for avulsion, HCA, and sham groups were 71.1% ± 8.3%, 68.4% ± 13.6%, and 68.8% ± 4.3%, respectively (Figure 3H, far left graph). Because of this similarity, fate choices in ventral horn were taken as representative of the entire spinal gray matter and were entered as such in all subsequent comparisons with fate choices in the meninges and white matter (see below).

In all animals examined, the pia adjacent to the inoculation sites contained large numbers of grafted NSCs. At those sites, differentiation pattern was different from parenchymal sites. For example, the rates of GFAP (+), astrocyte-like, NSC-derived cells were 30%–50% in the various experimental groups, and there were more cells persisting in a nestin (+) state (40%–53%) (Figure 3C, 3D, and 3G). At 6 mo, these rates were higher than those in ventral horn in all experimental groups (*p* < 0.05) (Figure 3H, center and far right graph). In sections that were dually labeled for human nestin and GFAP, the two phenotypes were expressed by distinct populations of NSC-derived cells, i.e. the vast majority of GFAP (+) cells were nestin (−) and nestin (+) cells were GFAP (−). Only 6%–11% of HNu (+) cells colocalized TUJ1 immunoreactivity at pial sites (Figure 3G). These rates are significantly lower compared to ventral horn and white matter (*p* < 0.05) (Figure 3H, far left graph).

The neuronal differentiation of NSCs that were dispersed in the ventral white matter, apparently by migration from the ventral horn inoculation sites, was not as prominent as in gray matter. At 6 mo postgrafting, percentages of dually labeled cell for TUJ1 and HNu in ventral white matter are 59.6% ± 6.3%, 57.8% ± 3.0%, and 59.9% ± 4.5% for avulsion, HCA, and sham treatments, respectively (Figure 3H, far left graph). Differences in TUJ1 (+) ratios between NSC-derived cells located in ventral white matter and in ventral horn are significant within the same treatment group (*p* < 0.05 for each one of the three groups). At the ventral white matter, percentages of GFAP (+) cells are 18.5% ± 5.6%, 20.0% ± 5.1%, and 16.1% ± 6.1% in the avulsion, HCA, and sham groups; in the avulsion group, GFAP differentiation rate is significantly higher than in ventral horn (*p* < 0.05) (Figure 3H, center graph). Nestin expression by HNu (+) cells is similar between ventral white matter and ventral horn (Figure 3H, far right graph).

In both parenchymal and meningeal locations, we observed infrequent HNu (+) cells that also expressed A2B5, a ganglioside antigen present in the common glial precursor O-2A, at 3 mo as well as 6 mo postgrafting. In 6-mo grafts, a population of smaller HNu (+) cells in the ventral horn expressed the mature oligodendrocyte marker adenomatus polyposis coli (APC) in the ventral horn (8.8% ± 4.1%, 8.9% ± 3.0%, and 9.0% ± 1.8 % in avulsion, HCA, and sham groups respectively), white matter (13.8% ± 2.6%, 12.2% ± 5.3%, and 12.0% ± 7.4%), and pia (6.4% ± 4.6%, 7.2% ± 3.3%, and 8.5% ± 1.9%). On confocal imaging, these cells have a thin cytoplasm and multiple APC (+) radial processes consistent with oligodendrocytic cytology (Figure 3E and 3F).

**Figure 4:**
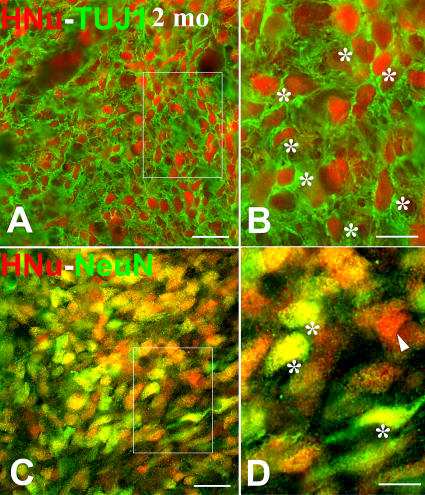
Differentiation of Human NSCs into Neurons after Transplantation into the Lumbar Spinal Cord of Normal Adult Sprague-Dawley Rats Outlined areas in (A) and (C) are enlarged in (B) and (D). All images illustrate the neuronal differentiation of NSCs two months postgrafting based on dual-label immunofluorescence for HNu (red) and a neuronal marker (green, representing TUJ1 and NeuN in [A and B], and [C and D], respectively). The predominance of double-labeled profiles in both (B) and (D) (indicated with asterisks) matches the avid neuronal differentiation of human NSCs in nude rats as illustrated in Figure 3. Single HNu-labeled profiles (D) are shown with arrowheads Scale bars: (A and B) 20 μm; (C and D) 10 μm.

In concert, the fate of grafted NSCs depends on location, i.e., the parenchymal microenvironment overall promotes a neuronal differentiation, and there is further inductive influence in this direction in the gray matter. The meningeal environment appears to facilitate astrocytic differentiation or to induce NSCs to remain in a nestin (+) state. In both locations, about one-tenth of surviving NSCs differentiate in the oligodendrocytic lineage. As proof of the concept that the predominantly neuronal differentiation of NSCs is not dependent on the athymic state of nude rats, we performed a small study in which we grafted the lumbar cord of normal rats. These animals were treated with FK506 to prevent xenograft rejection. Two months postgrafting, animals were prepared exactly as the nude rats and the neuronal differentiation of NSCs was explored with dual immunofluorescence using NeuN and TUJ1 as neuronal markers. As in the case of nude rats, the vast majority of NSCs had differentiated into neuronal cells (Figure 4).

### GABAergic and Cholinergic Neurotransmitter Phenotypes Expressed by Grafted NSCs

**Figure 5:**
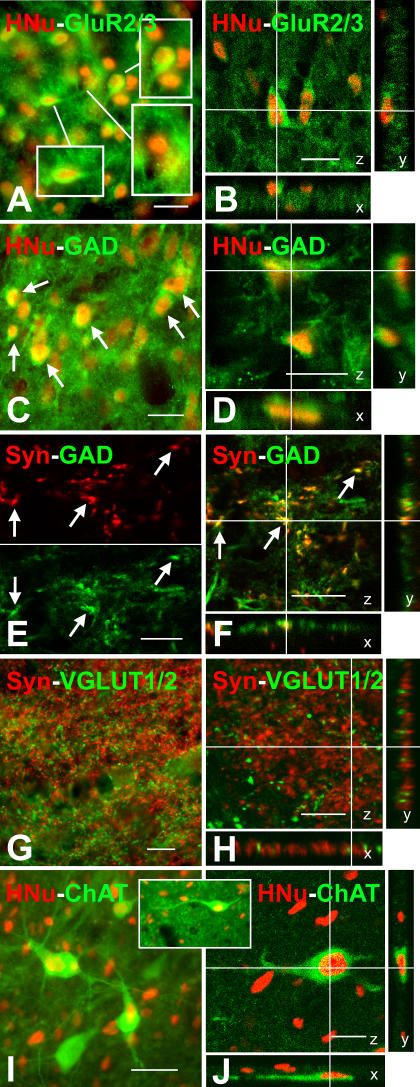
Neurotransmitter Differentiation of Grafted Human NSCs Photomicrographs (A–J) illustrate evidence of glutamatergic (A and B, G and H), GABAergic (C–F), and cholinergic (I and J) neurotransmission in NSC grafts. As in previous figures, confocal microscopy is used primarily to confirm the colocalization of two markers in the same cellular compartment along three planes of sectioning. (A and B) These sections, stained for HNu and the prevalent AMPA receptor epitope GluR2/3, show both cytoplasmic and synaptic staining by epifluorescence (A) or confocal (B) microscopy. Insets in (A) represent magnifications of indicated neurons in main image; top- and bottom-left insets show two medium-size HNu (+) cells with cytoplasmic immunoreactivity, whereas bottom-right inset illustrates a larger HNu (+) cell containing multiple GluR2/3 (+) boutons. (C and D) These sections are stained for HNu and the GABA-synthesizing enzyme GAD and visualized with epifluorescence (C) or confocal microscopy (D). Arrows in (C) indicate multiple HNu (+) cells with cytoplasmic GAD immunoreactivity. (E and F) Confocal microscopy of a field stained with both human Syn (red in single-channel image on top left, to label graft-derived terminals) and GAD (green in single-channel image on bottom left, to label GABAergic terminals) shows colocalization of the two proteins (yellow color in merged images in F) in multiple synaptic boutons. Nearly all graft-derived boutons are inhibitory (F). (G and H) These sections (G, epifluorescence; H, confocal) are stained for human Syn to label graft-derived terminals (red) and mixed VGLUT1/ VGLUT2 antibodies to label glutamatergic terminals in the field (green). Despite significant overlap and apposition of graft-derived and VGLUT1/2 (+) terminals (G), the two groups of terminals are separate (H). (I and J) These two sections were dually stained for: HNu and choline acetyltransferase (I and insert) epifluorescence; confocal microscopy (J); and show that some of the largest NSC-derived neurons express cholinergic phenotypes. These cells elaborate multiple primary dendrites (I and insert). (J) is the confocal image of the neuron in the inset. Scale bars: (A), (C), (G), (I) 20 μm; (B), (D–F), (H), (J) 10 μm.

Because of the predominant neuronal fate of grafted NSCs excitatory, inhibitory (GABA) and cholinergic neurotransmitter markers were examined in order to ascertain the degree of differentiation of cells into the neuronal lineage (Figure 5). Almost without exception, grafting sites were markedly enriched in metabotropic glutamatergic (Figure 5A and 5B) and GABAergic (Figure 5C and 5D) neurotransmitter markers. Individual bipolar NSC-derived cells were seen to express strong immunoreactivity for glutamate receptor subunit 2 and 3 (GluR2/3) and glutamate decarboxylase (GAD) and to be contacted by terminals enriched in these two neurotransmitter markers. Both GluR2/3 and GAD immunoreactivity first became apparent in grafting sites at 3 mo and persisted without appreciable changes at 6 mo postgrafting.

Examination of sections stained for HNu, GAD, and GluR2/3 showed a large number of NSC-derived neurons colocalizing both GAD and GluR2/3. To distinguish between GABAergic and glutamatergic phenotypes in the nerve terminals, we performed dual ICC for human-specific synaptophysin (Syn, to mark graft-derived terminals) and GAD or vesicular glutamate transporter type 1 and 2 (VGLUT1/2). GAD and VGLUT1/2 are sensitive and selective markers for GABAergic and glutamatergic neurons respectively [26,27], and terminal staining is especially robust in the spinal cord (Figure 5E and 5G). To label a maximal number of glutamatergic terminals, antibodies against VGLUT1 and VGLUT2 were used in combination. Large numbers of GAD and VGLUT1/2 (+) terminals were seen at the grafting sites, but fields of VGLUT1/2 (+) puncta only partially overlapped the graft (Figure 5G). In multiple fields in sections dually stained for human-specific Syn and GAD, we found that a majority of Syn (+) terminals colocalized GAD immunoreactivity by confocal microscopy (Figure 5F). In contrast, we observed no synaptic colocalization of human Syn with VGLUT1/2 immunoreactivity, despite dense apposition of individually labeled terminals (Figure 5H).

Using GAD immunoreactivity as a marker of GABAergic neurons, we counted HNu and GAD (+) profiles and calculated rates of dually labeled cells in the total population of HNu (+) cells at 6 mo, i.e. the longest survival time examined. At that time point, a significant percentage of HNu (+) cells in all three experimental groups (avulsion: 60.5% ± 0.47%; HCA lesion: 56.4% ± 3.19%; sham: 49.57% ± 4.04%) were also GAD immunoreactive. Frequency of differentiation did not vary significantly by type of treatment.

A very small percentage of HNu (+) cells (less than 1%), first appearing at 3 mo and consistently seen at 6 mo postgrafting, colocalized choline acetyltransferase immunoreactivity. Choline acetyltransferase (+) neurons were larger than other neuronal HNu (+) cells (15–25 μm in diameter) and displayed multipolar cytologies (Figure 5I and 5J).

These findings indicate that a majority of NSC-derived neurons develop and sustain stable bipolar cytologies and GABAergic phenotypes for at least six months after grafting. These cells are contacted by GABAergic terminals from other graft and host neurons and glutamatergic terminals from the host. A small, but consistent, percentage of graft-derived neurons evolve into larger multipolar neurons with cholinergic phenotypes.

### Evidence for Structural Integration of Human NSCs in Rat Spinal Cord: Axons and Synapses

**Figure 6:**
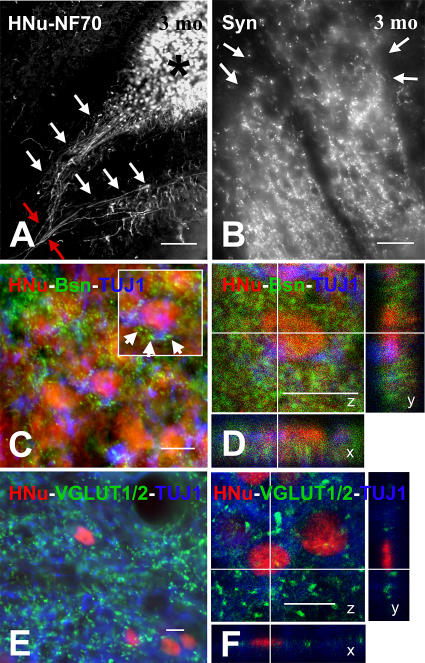
Maturation of Human NSC-Derived Neurons Based on the Elaboration of Axons, Synapses, and Innervation by Host Neurons (A) This photograph was taken through the ventral horn of a HNu/70 kDa neurofilament protein stained section 3 mo postgrafting and shows bundles of human 70 kDa neurofilament protein (+) axons (indicated with white arrows) originating in HNu (+) grafts (one indicated with an asterisk on top right) and coursing together (red arrows on bottom left) toward the ventral white matter. (B) This photograph shows an NSC graft in the ventral horn of a human Syn-stained section three months postgrafting. The sharp colocalization of Syn (+) puncta with the graft region (boundaries demarcated with arrows) is due to the selectivity of the antibody for human, but not rat, Syn protein. (C and D) These images (C, epifluorescence; D, confocal) were taken from triple-stained sections with HNu (red), TUJ1 (blue), and the presynaptic marker Bsn (green). The Bsn antibody used here recognizes rat and mouse, but not human, protein. (C) depicts a dense field of rat Bsn (+) terminals in proximity to HNu and TUJ1 (+) profiles. Examples of contacts between rat terminals and NSC-derived neurons are shown with arrowheads in the inset, which is a magnification of the profile at the center of the main image. The very large number of such terminals on NSC-derived cell bodies is best illustrated with confocal microscopy (D). (E and F) These photographs (E, epifluorescence; F, confocal) were taken from sections stained with HNu (red), TUJ1 (blue), and mixed VGLUT1/VGLUT2 antibodies (green) and show the innervation of HNu and TUJ1 (+) cells by glutamatergic terminals putatively originating in the host. Scale bars: (A) 80 μm; (B) 20 μm; (C–F) 10 μm.

By 3 mo postgrafting, HNu (+) neurons elaborate axons (Figure 6A) and synapses (Figure 6B) that can be specifically linked to graft origin with antibodies selective for human Syn and neurofilament proteins. Axons bundle in groups that traverse across the white matter (Figure 6A). Axon projections into the ventral root were rare or inconsistent. To evaluate the ability of HNu (+) neurons to integrate within the host circuitry, we labeled neural structures with graft- and host-selective markers. For example, to separate host from graft terminals, we used a monoclonal antibody for the presynaptic protein Bsn that selectively recognizes rat and mouse, but not human, epitopes. In sections stained for HNu (to establish graft origin), TUJ1 (to establish neuronal differentiation), and Bsn (to detect terminals from host rat axons), we found that HNu (+) and TUJ1 (+) cells in parenchymal locations were contacted by synaptic boutons of rat origin (Figure 6C and 6D), i.e., evidence that the host species (rat) innervates graft-derived (human) neuronal cells. Because graft-derived terminals are negative for markers of glutamatergic neurotransmission, VGLUT1/2 immunoreactivity can be used to differentiate between graft and host terminals. By combining ICC staining for HNu and TUJ1 (to mark graft-derived neurons) with ICC staining for VGLUT1/2 (to label most host-derived glutamatergic terminals), we found dense appositions between graft-derived neurons and host glutamatergic terminals (Figure 6E and 6F). In preparations stained for VGLUT1/2, GFAP, and HNu, we never observed such contacts between host terminals and graft-derived astrocytic profiles.

**Figure 7:**
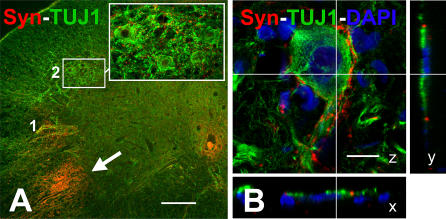
Innervation of Host Motor Neurons by Graft-Derived Nerve Cells as Shown on Sections Stained with Human Syn (Red) and TUJ1 (Green) and Studied with Epifluorescence or Confocal Microscopy Host motor neurons are depicted as large TUJ1 (+) cell bodies, and NSC-derived terminals are labeled with human Syn antibodies. (A) This epifluorescence image shows the site of the original graft (arrow in lower left) and two synaptic fields with host motor neuron pools marked as (1) and (2), with respectively higher and lower density of synaptic appositions. The low-density field (2) is further enlarged in the inset. (B) This confocal image shows, in great detail, a large number of somatic and dendritic terminals from graft-derived nerve cells on a host motor neuron. Scale bars: (A) 200 μm; (B) 20 μm.

Conversely, preparations stained with human-specific Syn and TUJ1 or choline acetyltransferase revealed dense terminal fields of boutons apposed to host neurons, including large and small motor neurons both on the side of grafting as well as the contralateral side. Large numbers of host motor neurons were seen to be contacted in such a fashion by graft-specific terminals (Figure 7A and 7B). Treatment variance (avulsion, HCA lesion, or sham treatment) did not seem to affect the presence or absence of such dense contacts. In sections stained for human Syn and GFAP, we did not observe any appositions between graft-derived terminals and astrocytes.

In summary, NSC-derived neurons do not only develop differentiated neurotransmitter phenotypes in the adult spinal cord, but also elaborate axons and synaptic specializations and form synaptic contacts with host spinal cord neurons.

## Discussion

### Generalities

Our findings indicate a large-scale differentiation and some structural integration of human NSCs grafted into the normal and injured spinal cord of T cell deficient (nude) rats. Under the present experimental conditions, human NSCs survive well with limited further mitotic activity and migrate extensively for at least six months postgrafting. Although the vast majority of NSCs in the spinal cord parenchyma take on a neuronal fate, the meningeal environment is associated with astrocytic phenotypes or the persistence of grafted NSCs in a perpetual nestin (+) state.

A majority of NSC-derived neurons in spinal cord parenchyma have bipolar cytologies and GABAergic phenotypes for at least six months after grafting and receive GABAergic innervation from other graft and host neurons, in addition to glutamatergic innervation from the host. A small percentage of graft-derived neurons evolve into larger multipolar neurons with cholinergic phenotypes. NSC-derived neurons elaborate axons and synaptic specializations and appear to engage in reciprocal innervation with host spinal cord neurons. Importantly, when immunological obstacles are overcome, rodent models are quite suitable for the preclinical evaluation of human NSCs as tools for repair of the injured spinal cord.

### Survival and Migration

The 3- to 4-fold increase in NSC numbers postgrafting, although not of the magnitude or rate to cause tumors, persisted during the period of active neuronal differentiation. Because it is implausible that the differentiation and dividing pools of NSCs are the same cell population, we hypothesize that the subset of grafted NSCs that persists in a nestin (+) state, for example NSC-derived cells located near the pial surface, is the one that gives rise to additional neuronal lineage, possibly on an ongoing basis. These cells may persist in a stem cell state either because of accidental initial placement near the pial environment or via active migration and tropism to pial sites. These are complex phenomena that require NSC-host signaling and require further investigation.

The migratory spread of human NSCs was especially evident at three and six months, but a migratory disposition was ascertained by Dcx immunoreactivity as early as three weeks postgrafting. Although most HNu (+) cells remained within the gray matter, there was also colonization of the white matter. A consistent phenomenon was the early establishment of linear pathways of migration from the initial graft into the white matter perpendicular to the spinal cord surface, which often appeared to extend all the way to the pia. In 4′,6-diamidino-2-phenylindole-counterstained preparations, it was apparent that these migratory pathways were associated with microvessels. The generality of this phenomenon invites further exploration of the role of microvessels as guiding structures for NSC migration and of the role of specific cells in the host vasculature as generators of tropic and other signals for grafted NSCs.

### Differentiation and Fate Choices

Neuronal differentiation of NSC grafts in the adult spinal cord has been the exception, rather than the rule, in the literature [6–9,11]. The relative success in the neuronal differentiation of NSCs when these cells were grafted into the developing spinal cord [28] is consistent with the idea that essential inductive signals are present in the immature but perhaps not the adult spinal cord [5,29]. The substantial neuronal differentiation of embryonic stem cell/embryonic body-derived NSCs in the adult spinal cord achieved by McDonald and colleagues [10] was the first indication that the adult spinal cord environment may allow neuronal differentiation under certain conditions. More promising results were seen when human neuroteratocarcinoma cells or committed neuronal precursors from rat spinal cord were grafted in the ischemic spinal cord [12]. Survival of grafted neurons in this report was very low, perhaps because of immunological rejection, but graft-derived neurons assumed GABAergic phenotypes and showed robust axonal growth.

The extensive neuronal differentiation of the NSC preparation used in the present study may be due to several reasons, including species of NSC origin and culture method. For example, human NSCs may have a greater pluripotentiality compared to rodent NSCs [30]. Also, the fact that ~ 5% of human NSCs used here express PSA-NCAM prior to grafting implies that a small portion of grafted cells had already made a neuronal lineage choice. However, this preinoculation choice of a small minority of grafted NSCs cannot account for the massive neuronal differentiation of the graft that is bound to have derived through multiple-step differentiation postgrafting. Technical matters pertaining to the preparation of cells, e.g., their propagation in monolayer cultures that prevents a harsher treatment prior to their suspension for grafting could also have played a role in the outcome.

The robust differentiation of NSCs in this study is inconsistent with the general notion that the adult cord environment is constitutively unfavorable to the survival and differentiation of NSCs [5,29]. At the very least, our work demonstrates that graft-intrinsic factors may be just as important as the host microenvironment in determining the in vivo differentiation of grafted NSCs. The T cell-deficient state of the experimental animals used here is unlikely to have influenced the grafting outcome. In addition to the successful grafting of human NSCs in nude rats, we have achieved excellent survival and neuronal differentiation of human NSC grafts in completely normal rats as well as in SOD1-G93A transgenic rats and mice [31,32]. Cummings and colleagues have recently published a report showing that a quarter of human fetal brain-derived NSCs differentiate into neurons in the contused spinal cords of NOD-*scid* mice [33]. In another study published while this paper was in review [34], more than 50% of mouse NSCs grafted into the sindbis virus-infected spinal cord differentiated into neurons, a large percentage of which showed phenotypes and cytologies of spinal motor neurons. Although there is substantial variety among these models and only one other study used human NSCs [33], the gist of this recent work is that neuronal differentiation of NSC grafts is certainly possible in the mature spinal cord.

Our findings also show that local factors can influence the fate choice of grafted NSCs, i.e., many more NSCs turn into astrocytes or remain nestin (+) when located close to the meninges (pia). The gray matter may exert additional inductive influences in the direction of neuronal differentiation and certain lesions, i.e., root avulsions, appear to facilitate an astrocytic fate choice. These patterns are welcome evidence of NSC plasticity and suggest that signals from the host microenvironment play significant roles in the differentiation choices of these cells in vivo. However, before various preparations of cells are directly compared in the same animal model, it is premature to draw generic conclusions about differentiation trends of human NSCs in the adult mammalian cord.

An unexpected finding in this paper was the measurable differentiation of human NSCs into mature oligodendrocytes six months after transplantation, especially given the low preference of these cells for oligodendrocyte fate choice in vitro. This is yet another indication of an important role of the mature spinal cord environment in inducing relatively immature precursors into diverse differentiation pathways. Oligodendrocyte differentiation was the predominant fate of a preparation of brain-derived human NSCs that was recently grafted into the contused spinal cord of NOD-*scid* mice [33].

### Neurotransmitter Identification and Axonal Growth

The predominant neurotransmitter phenotype of differentiated NSCs is GABAergic, although a sizeable minority of these cells acquires cholinergic phenotypes. It has been reported that “priming” of human NSCs prior to grafting may advance them more fully to cholinergic fates postgrafting [35,36], but the likely advanced maturation state of these cells pregrafting and unknown survival rates and grafting efficiency does not allow direct comparisons with the results of this study. GABAergic and cholinergic neurotransmitter signatures appear to belong to neurons with different cytologies, i.e., GABAergic neurons have smaller bipolar cytologies whereas cholinergic neurons are larger and multipolar. However, all shapes and sizes between these two types have been encountered and, in a few cases of grafts growing outside the spinal cord parenchyma, we have seen a continuous pattern of differentiation in colonies in which GABAergic neurons predominate in the periphery and cholinergic neurons at the center. Therefore, it is unlikely that these two phenotypes belong at the end points of distinct differentiation lineages and it is possible that some GABAergic neurons could turn into cholinergic nerve cells given the proper instructive signals. Another argument against the idea of the GABAergic phenotype as an end-point fate is the fact that a majority of HNu (+) cells in the spinal cord extend long axons, whereas GABAergic spinal cord neurons in mature animals are interneurons [37,38].

A substantial degree of elongation of NSC-derived axons was observed within the spinal cord parenchyma. This observation suggests very little, if any, inhibitory effect from the host in the elaboration and elongation of these axons. The myelin-associated glycoprotein and the oligodendrocyte-myelin glycoproteins NOGO A, B, and C have been shown to inhibit axonal regeneration [39], although neurotrophic factors can antagonize these effects by increasing intracellular cAMP levels [40,41]. The lack of marked axonal growth inhibition seen in our cases may be due to several factors, one of which may be the minimally invasive nature of the lesions employed here with little disruption of the blood–brain barrier and absence of glial scarring. It has been previously demonstrated that transplantation techniques that minimize direct injury to spinal cord parenchyma lead to successful axonal growth by limiting the adverse impact of the reactive extracellular matrix at the lesion site and by preventing cavitation and astrocyte transformation/migration [42,43]. Successful axonal regeneration without the use of supplemental growth factors or manipulations of cAMP levels has been reported from additional groups using grafts of olfactory ensheathing [44] and developing human spinal cord [45] cells in models of spinal cord injury.

### Structural Integration of Graft with Host and Conclusions

The partial anatomical integration of grafted cells with host neurons via the establishment of contacts observed here has important theoretical and practical implications. Although this is a novel phenomenon and the molecular mechanisms are unknown, a number of trophic and cell–cell recognition transcripts shown to be expressed by cultured human NSCs are likely to play significant roles in this process. These protein signals are expressed and released in amounts and gradients that allow highly conserved tropic and trophic interactions to occur irrespective of age of the host, or the fact that host and graft belong to different species. Based on our PCR data on human NSCs in culture, these proteins include: the prototypical glial-derived growth factor glial cell line-derived neurotrophic factor and the neurotrophin BDNF, i.e. potent trophic factors for motor neurons that can account for the marked ability of human NSCs to attract host motor axons [46–48]; the NRGs 1, 2, and 3, i.e., critical glial trophic signals that may also guide host axons and promote host–graft integration [49–55]; the growth hormone-like peptide IGF-1; and angiogenic factors such as fibroblast growth factor and VEGF. Besides the neurotrophic effects of VEGF and IGF-1 [56–58], VEGF may also play a role in migration and position patterning of developing motor neurons [59]. Interestingly, IGF-1 may work in tandem with BDNF to promote neuronal precursor differentiation [60], and this interaction may be particularly relevant for the findings of this study.

In conclusion, NSCs from human spinal cord propagated in vitro can be grafted successfully in the adult rat spinal cord under a variety of experimental conditions and show marked differentiation into projection neurons that appear to engage in circuit formation with the host irrespective of species differences. These outcomes indicate that, with successful suppression of host immune rejection of the graft, rodent models can be used for the preclinical evaluation of human NSCs as cell support or replenishment tools to a much larger extent than previously anticipated. It appears that cellular and molecular factors that signal synaptic contact formation are remarkably conserved within the mammalian class, despite the fact that spatial and temporal patterning of developmental events may differ among species. Together, our findings add weight to the argument that restoration of spinal cord circuitry in traumatic and degenerative diseases may be a realistic goal that needs to be further refined at the preclinical stage prior to its eventual consideration for clinical applications.

## Abbreviations

APC: adenomatus polyposis coli
bFGF: basic fibroblast growth factor
Bsn: Bassoon
Dcx: doublecortin
GABA: gamma-aminobutyric acid
GAD: glutamate decarboxylase
GFAP: glial fibrillary acidic protein
GluR2/3: glutamate receptor subunit 2 and 3
HCA: L-homocysteic acid
HNu: human nuclear antigen
ICC: immunocytochemistry
IGF-1: insulin-like growth factor-1
IgG: immunoglobulin G
NRG: neuregulin
NSC: neural stem cell
Syn: synaptophysin
VEGF: vascular endothelial growth factor
VGLUT: vesicular glutamate transporter
